# Allosteric modulation of the fish taste receptor type 1 (T1R) family by the extracellular chloride ion

**DOI:** 10.1038/s41598-023-43700-y

**Published:** 2023-09-28

**Authors:** Ryusei Goda, Soichi Watanabe, Takumi Misaka

**Affiliations:** 1 Department of Applied Biological Chemistry, Graduate School of Agricultural and Life Sciences, The University of Tokyo, 1-1-1 Yayoi, Bunkyo-ku, Tokyo, 113-8657 Japan; 2 Department of Aquatic Bioscience, Graduate School of Agricultural and Life Sciences, The University of Tokyo, Tokyo, Japan

**Keywords:** Molecular biology, Cell signalling

## Abstract

Many G protein-coupled receptors (GPCRs) are allosterically modulated by inorganic ions. Although the intraoral ionic composition of the oral cavity varies depending on the living environment and feeding behavior, little is known about whether and how it affects the function of taste receptor type 1 (T1R), a member of the class C GPCR family. Here, we report that chloride ions allosterically modulate the functions of specific fish T1Rs, namely, mfT1R2a/mfT1R3 and zfT1R2a/zfT1R3. Site-directed mutagenesis revealed mfT1R2a K265, which lies in the extracellular domain of mfT1R2a, to be as a critical residue for the modulation of mfT1R2a/mfT1R3 by Cl^−^. However, this residue is not conserved in zfT1R2a, and the introduction of the key residue at the corresponding site of another T1R, mfT1R2b, did not confer Cl^−^ susceptibility. These results indicate the variability of the determinants of Cl^−^ susceptibility.

## Introduction

In vertebrates, sweet, umami, and bitter tastes are mediated by the G protein-coupled receptor (GPCR) family^1^. Taste receptor type 1 (T1R), a class C GPCR with a large extracellular domain called the Venus flytrap domain (VFTD), functions as a heterodimer of T1R1/T1R3 or T1R2/T1R3 and detects L-amino acids, 5'-ribonucleotides, or sugars^2–6^. While the cleft region within the VFTD is considered an orthosteric binding site of T1Rs^7–9^, several allosteric modulators that modulate receptor activities by binding outside the orthosteric sites have also been reported. In particular, multiple allosteric modulators of the human sweet taste receptor (hT1R2/hT1R3) have been identified through the exploration of noncaloric sweeteners, such as SE-2 and FEMA4774 as positive allosteric modulators (PAMs)^10,11^ and lacitisole and 2,4-DP as negative allosteric modulators (NAMs)^12,13^. In contrast, inosine 5'-ribonucleotides, S807, and methional affect the umami taste receptor (T1R1/T1R3) in an allosteric manner^7,14^, although they function differently as PAMs/NAMs or agonists/modulators depending on the species^5,14^.

It has also been found that substances other than low molecular weight chemical compounds can act as allosteric modulators of GPCRs. Reportedly, many GPCRs are susceptible to modulation by several types of inorganic ions^15,16^, and class C GPCRs are no exception. For example, Ca^2+^, an agonist of the calcium-sensing receptor (CaSR)^17^, regulates the function of metabotropic glutamate receptors (mGluRs)^18,19^. Among anions, Cl^−^ acts as a PAM for mGluRs and CaSR^20–22^, while SO_4_^2−^ and PO_4_^3−^ allosterically inhibit CaSR^23,24^.

In contrast, regarding the ionic composition of the human oral cavity, the concentrations of Na^+^ and Cl^−^ in unstimulated human saliva are approximately 5–15 mM and 10–20 mM, respectively, and these values could change periodically^25–27^. In aquatic fish, on the other hand, the intraoral ionic composition varies dramatically, depending on the surrounding environment. The concentrations of Na^+^ and Cl^−^ in river water are generally ~ 2 mM and ~ 1 mM, respectively, although there are regional and seasonal differences^28,29^, whereas both ions exists in seawater at concentrations over 400 mM^30^. In addition, the concentrations of these ions (100–200 mM^30^) in the extracellular body fluid of animals, which may have been preyed upon by both humans and fish, are distinct from the values listed above, indicating drastic temporal fluctuations in the intraoral ionic conditions when eating food.

Nonetheless, only a few studies focusing on the interactions between T1Rs and inorganic ions have been conducted^31,32^. Meanwhile, the crystal structure of the extracellular ligand-binding domains of T1R2a, a subtype of T1R2, and the T1R3 heterodimer from medaka fish (mfT1R2a/mfT1R3) implied the existence of Na^+^ and Cl^−^ binding to the receptor^8^. Additionally, a recent report indicated, through structural, biophysical, and physiological analyses, that Cl^−^ acts as an agonist at sub-mM to low-mM concentrations by binding to a well-conserved chloride ion-binding site within the extracellular domain of the T1R3 subunit^33^.

In this study, we investigated the functional role of Cl^−^ on several T1Rs through a cellular functional assay and showed that Cl^−^ actually works as an allosteric modulator of particular fish T1Rs at concentrations of tens of mM. Through site-directed mutagenesis, a previously elucidated Br^−^ binding site within mfT1R2a^33^ was confirmed to be a key residue for allosteric modulation by Cl^−^. However, the residue is not conserved in zebrafish T1R2a (zfT1R2a), whose sensitivity to L-Pro when coexpressed with zfT1R3 was susceptible to modulation by Cl^−^. In addition, the introduction of the residue at the corresponding site of another T1R, mfT1R2b, did not confer Cl^−^ susceptibility. These results suggest complicated mechanisms determining the Cl^−^ susceptibility of T1Rs.

## Results

### Response of the taste receptors under low Cl^−^ conditions

First, the Cl^−^ susceptibility of wild type (WT) T1Rs of human and fish origin (hT1R2/hT1R3, mfT1R2a/mfT1R3, mfT1R2b/mfT1R3, mfT1R2c/mfT1R3, zfT1R2a/zfT1R3, and zfT1R2b/zfT1R3) was evaluated. Because of unstable cellular responses to agonists when T1R-expressing cells were maintained in buffers with 5 mM Cl^−^ or lower, in our experimental conditions, the lowest concentration of Cl^−^ in the buffer was set at 10 mM. The agonists applied to each receptor were selected according to our previous publications^6,9,34^. It should be noted that although the fish T1Rs commonly respond to L-amino acids, there are differences in their ligand profiles. While mfT1R2a/mfT1R3 and mfT1R2b/mfT1R3 are broadly tuned receptors, mfT1R2c/mfT1R3 is a narrowly tuned receptor highly sensitive and specific to L-Pro^6,8,34^. Both zfT1R2a/zfT1R3 and zfT1R2b/zfT1R3 respond to several L-amino acids, though the sensitivity of zfT1R2a/zfT1R3 is higher than zfT1R2b/zfT1R3^6,34^.

**Figure 1:**
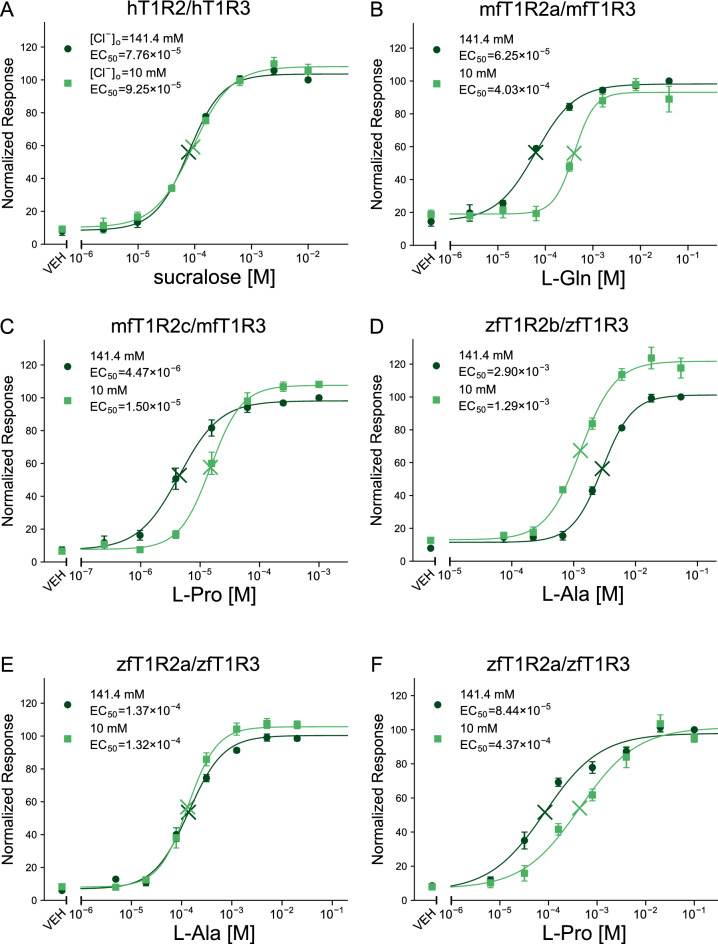
Different susceptibilities of T1Rs to extracellular anionic composition. Dose‒response curves of stable cell lines expressing (**A**) hT1R2/hT1R3 to sucralose, (**B**) mfT1R2a/mfT1R3 to L-Gln, (**C**) mfT1R2c/mfT1R3 to L-Pro, (**D**) zfT1R2b/zfT1R3 to L-Ala, (**E**) zfT1R2a/zfT1R3 to L-Ala, or (**F**) zfT1R2b/zfT1R3 to L-Pro, with G16gust44. The raw data were normalized to the mean response against the highest concentration of ligands obtained in high Cl^−^ buffer in each experiment. Data are shown as the mean ± standard error of the mean (SEM, n = 3–5 independent experiments performed in duplicate or triplicate). Cross marks in the graph represent EC_50_ values. VEH: responses against the buffer.

The sensitivity of hT1R2/hT1R3-expressing cells to sucralose, D-tryptophan, and cyclamate was comparable between high (141.4 mM) Cl^−^ buffers and 10 mM Cl^−^ buffers (Figs. 1A, S1A,B), indicating that high concentrations of extracellular gluconate, as well as low concentrations of extracellular Cl^−^ ([Cl^−^]_o_, ≥ 10 mM), did not strikingly disturb Ca^2+^ mobilization nonspecifically under our experimental conditions. In contrast, the sensitivity of several fish T1Rs was actually affected by the anionic composition of the buffers. In 10 mM Cl^−^ buffer, the EC_50_ values of mfT1R2a/mfT1R3 to L-Gln and L-Ala were approximately 6.5 and 7 times higher than those in high Cl^−^ buffer (Figs. 1B, S1C). mfT1R2c/mfT1R3 was less sensitive to the composition, showing an approximately threefold increase in EC_50_ values in 10 mM Cl^−^ buffer compared to that in high Cl^−^ buffer (Fig. 1C), whereas the sensitivity of mfT1R2b/mfT1R3 to L-Ala and L-Pro was hardly affected (Fig. S1D,E). For zfT1R2b/zfT1R3, conversely, the EC_50_ values for L-Ala and L-Pro in 10 mM Cl^−^ buffer were approximately half of those in high Cl^−^ buffers (Figs. 1D, S1F). Notably, the effect of the anionic composition of the buffers on zfT1R2a/zfT1R3 was agonist dependent; while its EC_50_ value for L-Ala was not influenced, that for L-Pro was approximately 5 times higher in 10 mM Cl^−^ buffer (Fig. 1E,F).

**Figure 2:**
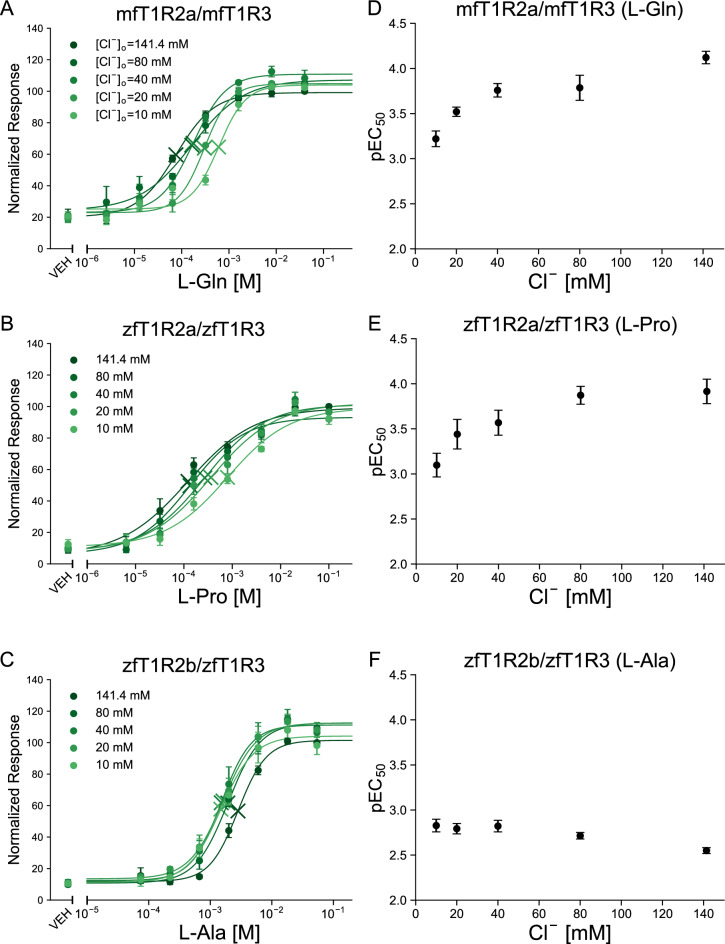
The relationships between extracellular Cl^−^ concentration and the sensitivity of fish T1Rs. (**A**–**C**) Dose‒response relationships of (**A**) mfT1R2a/mfT1R3 to L-Gln, (**B**) zfT1R2a/zfT1R3 to L-Pro, or (**C**) zfT1R2b/zfT1R3data dat to L-Ala obtained at 10 mM, 20 mM, 40 mM, 80 mM, and 141.4 mM [Cl^−^]_o_. Data are shown as the mean ± SEM (n = 3 independent experiments performed in triplicate). VEH: responses against the buffer. (**D**–**F**) The negative logarithm of the EC_50_ values expressed in mol/L units (pEC_50_) calculated in (**A**)–(**C**) were plotted against [Cl^−^]_o_. Error bars indicate SEM.

Hereafter, four receptors (mfT1R2a/mfT1R3, mfT1R2c/mfT1R3, zfT1R2a/zfT1R3, and zfT1R2b/zfT1R3) whose EC_50_ ratios between high Cl^−^ buffer and 10 mM Cl^−^ buffer were less than 1/2 or more than 2 were further examined. To further investigate the modulatory characteristics of extracellular Cl^−^, functional assays were conducted in buffers containing intermediate concentrations of Cl^−^. mfT1R2a/mfT1R3 and zfT1R2a/zfT1R3 showed clear reductions in sensitivity at [Cl^−^]_o_ below 80 mM (Fig. 2A–E). On the other hand, a decrease in the sensitivity of zfT1R2b/zfT1R3 was observed in the range from 40 to 141.4 mM [Cl^−^]_o_ (Fig. 2C,F). Notably, the EC_50_ value of mfT1R2c/mfT1R3 for L-Pro remained almost unchanged in the range from 10 to 80 mM [Cl^−^]_o_ (Fig. S2), which indicates the primary contribution of gluconate and/or only a modest contribution of Cl^−^ to its sensitivity. Therefore, mfT1R2c/mfT1R3 was excluded from the subsequent experiments.

These results suggest that Cl^−^ can differentially modulate the function of some fish T1Rs, although the impact of gluconate species cannot be completely excluded.

### Chloride, rather than gluconate, allosterically modulates mfT1R2a/mfT1R3 and zfT1R2a/zfT1R3

To confirm whether Cl^−^, not gluconate^−^, serves as an allosteric modulator for fish T1Rs, NaCl was applied alone or with agonists to the cells maintained in 10 mM Cl^−^ buffer without changing the gluconate concentration before and after ligand application, as explained in the *Materials and methods* (detailed ion compositions are also listed in Supplemental Tables S1 and S2). In addition, sodium gluconate was used instead of NaCl to account for the osmotic pressure and cation composition.

When NaCl was coapplied with 150 µM sucralose, hT1R2/hT1R3 showed no significant difference in response intensity up to 60 mM [Cl^−^]_o_ (compared to the response to sucralose at 10 mM [Cl^−^]_o_, one-way analysis of variance (ANOVA) followed by Dunnett’s test), although the responses decreased at higher concentrations (Fig. S3A). This decrease can be explained by an increase in osmotic pressure and/or extracellular sodium concentration ([Na^+^]_o_) because the responses showed a similar reduction when Na-gluconate was coapplied with sucralose (Fig. S3B). These results are consistent with those of previous experiments, in which the response of hT1R2/hT1R3 to sucralose was almost unaffected by lowering [Cl^−^]_o_ (Fig. 1A). In addition, neither NaCl nor Na-gluconate alone elicited cellular responses under these conditions (Fig. S3B).

**Figure 3:**
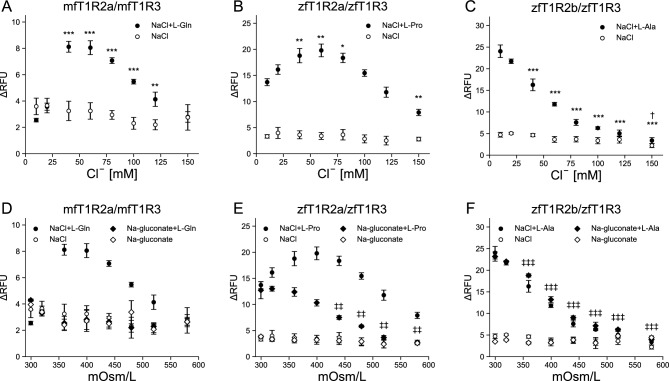
The modulatory action of Cl^−^ applied to the cells maintained in 10 mM Cl^−^ buffer. (**A**–**C**) The responses of cells expressing (**A**) mfT1R2a/mfT1R3, (**B**) zfT1R2a/zfT1R3, or (**C**) zfT1R2b/zfT1R3 with G16gust44. The cells were maintained in 10 mM Cl^−^ buffer and stimulated with NaCl solution to obtain the final ionic composition, as shown in Supplementary Table S1, with or without corresponding agonists (200 µM L-Gln for mfT1R2a/mfT1R3, 160 µM L-Pro for zfT1R2a/zfT1R3, or 2 mM L-Ala for zfT1R2b/zfT1R3). The responses were plotted against the final [Cl^−^]_o_ concentrations. (**D**–**F**) Na-gluconate solution was also applied to the cells expressing (**D**) mfT1R2a/mfT1R3, (**E**) zfT1R2a/zfT1R3, or (**F**) zfT1R2b/zfT1R3 to achieve the final ionic composition as shown in Supplementary Table S2. The responses to Na-gluconate solution were plotted along with the responses to the NaCl solution shown in (**A**)–(**C**) against the final osmotic pressure. Three independent experiments were conducted for each receptor, and representative results are shown as the mean ± SEM (n = 3 replicates). Significant differences were analyzed using a one-way analysis of variance (ANOVA) followed by Dunnett’s test (*p < 0.05, **p < 0.01 and ***p < 0.001 for vehicle vs NaCl + agonists; †*p* < 0.05 for vehicle vs NaCl; ‡*p* < 0.05, ‡‡*p* < 0.01 and ‡‡‡*p* < 0.001 for vehicle vs Na-gluconate + agonists). ∆RFU: Delta relative fluorescent units. VEH: responses against the 10 mM Cl^−^ buffer with or without agonists.

In the case of mfT1R2a/mfT1R3 and zfT1R2a/zfT1R3, their responses to a fixed concentration of L-Gln (200 µM) or L-Pro (160 µM), respectively, were enhanced with an increase in the final [Cl^−^]_o_ up to 60 mM, whereas NaCl alone did not elicit their responses (Fig. 3A,B). The reduction in the responses at higher [Cl^−^]_o_ is likely due to osmotic pressure, as in the case of hT1R2/hT1R3 (Fig. S3A,B). Coapplication of Na-gluconate instead of NaCl with the agonists, however, did not enhance the response intensity (Fig. 3D,E). These results indicate that Cl^−^ actually affects the sensitivity of mfT1R2a/mfT1R3 and zfT1R2a/zfT1R3, and that Cl^−^ acts as a PAM rather than an agonist under our experimental concentration ranges, contrary to a recent publication^33^.

Conversely, the responses of zfT1R2b/zfT1R3 to a fixed concentration of L-Ala (2 mM) decreased with increasing NaCl concentrations (Fig. 3C). However, similar phenomena were observed even when Na-gluconate was coapplied (Fig. 3F), making it difficult to discriminate between the potential suppressive effect of Cl^−^ and osmotic pressure. Therefore, it remains unclear whether Cl^−^ or gluconate affects the function of zfT1R2b/zfT1R3.

### The conserved site within T1R3 is not the key to the modulatory effect of extracellular Cl^−^ on mfT1Rs

The crystal structures of the VFTD of the mfT1R2a/mfT1R3 heterodimer showed the existence of a Cl^−^ binding site within the T1R3 subunit^8,33^, and this site is widely conserved among T1R3s of various species as well as other class C GPCRs^20,22,33^. To verify the contribution of this site, hereinafter called “site X”, to the observed allosteric modulation of T1Rs by extracellular Cl^−^, five cell lines expressing hT1R2/hT1R3 T102E, mfT1R2a/mfT1R3 T105E, mfT1R2b/mfT1R3 T105E, mfT1R2c/mfT1R3 T105E, and zfT1R2b/zfT1R3 T110E, all of which were expected to prevent Cl^−^ from binding to the site, were constructed.

**Figure 4:**
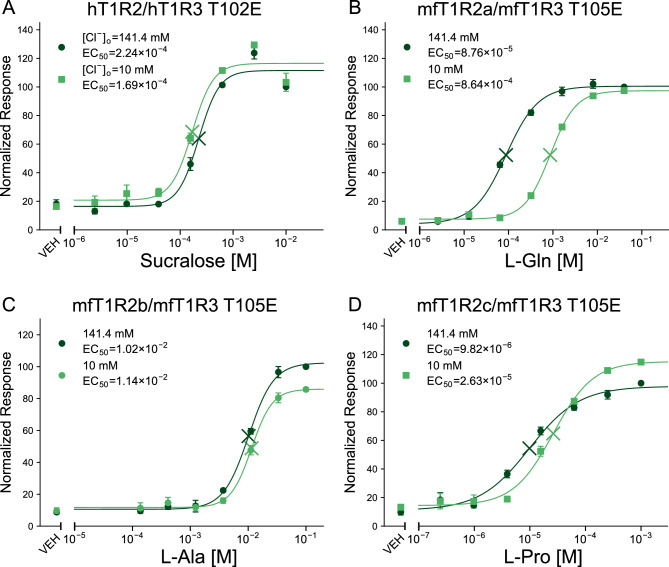
Site X does not affect the susceptibility of T1Rs to Cl^−^. Dose‒response curves of stable cell lines expressing (**A**) hT1R2/hT1R3 T102E to sucralose, (**B**) mfT1R2a/mfT1R3 T105E to L-Gln, (**C**) mfT1R2b/mfT1R3 T105E to L-Ala, or (**D**) mfT1R2c/mfT1R3 T105E to L-Pro, with G16gust44. Data are shown as the mean ± SEM (n = 3–4 independent experiments performed in duplicate or triplicate). Cross marks in the graph represent EC_50_ values. VEH: responses against the buffer.

As zfT1R2b/zfT1R3 T110E did not show obvious responses in high Cl^−^ buffer (Fig. S4A), it was excluded from subsequent experiments. All other mutants showed buffer composition susceptibility similar to that of the wild type (Fig. 4). The sensitivities of hT1R2/hT1R3 T102E and mfT1R2b/mfT1R3 T105E were barely affected by [Cl^−^]_o_ (Fig. 4A,C). In contrast, the EC_50_ values of mfT1R2a/mfT1R3 T105E and mfT1R2c/mfT1R3 T105E were clearly higher in 10 mM Cl^−^ buffer, although the difference was greater in mfT1R2a/mfT1R3 T105E than in mfT1R2c/mfT1R3 T105E (Fig. 4B,D), which was consistent with the trend observed in the wild type (Fig. 1B,C). These results suggest that site X within the T1R3 subunit is not critical for allosteric modulation by extracellular Cl^−^.

### The Br^−^ binding site in mfT1R2a is one of the key sites for the modulatory effect of extracellular Cl^−^ on T1Rs

**Figure 5:**
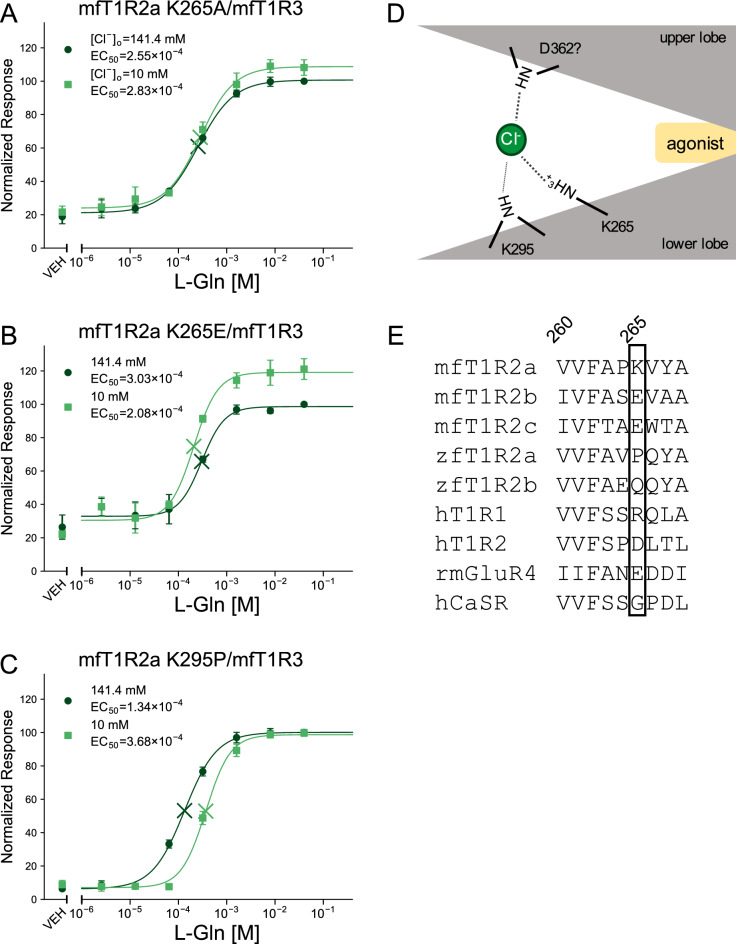
The mutation in site Y disturbed the action of Cl^−^ as PAM on mfT1R2a/mfT1R3. (**A**–**C**) Dose‒response curves of stable cell lines expressing (**A**) mfT1R2a K265A/mfT1R3, (**B**) mfT1R2a K265E/mfT1R3, or (**C**) mfT1R2a K295P/mfT1R3 to L-Gln, with G16gust44. Data are shown as the mean ± SEM (n = 3–4 independent experiments performed in duplicate or triplicate). Cross marks in the graph represent EC_50_ values. VEH: responses against the buffer. (**D**) Schematic illustration of the mechanism by which Cl^−^ acts as PAM on mfT1R2a/mfT1R3. The labels “upper lobe” and “lower lobe” indicate the upper and lower lobes within the Venus flytrap domain of mfT1R2a. (**E**) Amino acid sequence alignment of seven T1Rs from medaka fish, zebrafish, and humans; rat mGluR4; and human CaSR at site Y.

As site X was not primarily responsible for the modulation by Cl^−^, as described above, we then focused on the recently proposed Br^−^ binding site within mfT1R2a VFTD^33^, hereinafter called “site Y”. Therefore, to prevent anion binding due to ionic interactions, mfT1R2a K265, the critical residue at site Y, was mutated to alanine or glutamate to abolish the positive charge on the side chain or to change it to a negative charge, respectively. The reduction in sensitivity from high Cl^−^ buffer to 10 mM Cl^−^ buffer was almost eliminated in both mutants (Fig. 5A,B), suggesting that site Y is critical for the modulation of mfT1R2a/mfT1R3 by extracellular Cl^−^.

To further clarify the molecular basis of allosteric modulation by Cl^−^, several other mutants of residues within the lower lobe of mfT1R2a VFTD near K265 were created (Figs. 5C, S5A–C). In addition to alanine, mutations to proline were also examined to validate the contribution of main-chain amides. Among the mutants tested, the function of mfT1R2a N294A/mfT1R3 and mfT1R2a N294P/mfT1R3 was severely disturbed and their susceptibility to Cl^−^ could not be evaluated (Fig. S4B,C). Among the other mutants, mfT1R2a K295P/mfT1R3 showed weakened susceptibility to Cl^−^ (Fig. 5C), whereas mfT1R2a K295A/mfT1R3 displayed a similar reduction in sensitivity to WT mfT1R2a/mfT1R3 in 10 mM Cl^−^ buffer (Fig. S5A), suggesting that the main-chain amide of mfT1R2a K295, rather than its side chain, plays a supplementary role in its interaction with Cl^−^.

As Cl^−^ is suggested to function as a PAM of mGluRs by binding to the upper and lower lobes to stabilize the closed active conformation^20,21^, we also attempted to identify the key residues in the upper lobe of mfT1R2a around site Y. Although the main-chain amide of mfT1R2a D362 seems to be the primary candidate for the residue based on the structural information^33^, we could not directly verify this hypothesis, as mfT1R2a D362P/mfT1R3 did not show obvious responses in high Cl^−^ buffer (Fig. S4D). However, all other mutations in the upper lobe of mfT1R2a, except for mfT1R2a R70A/mfT1R3, which was also a loss-of-function mutant (Fig. S4E), sustained Cl^−^ susceptibility similar to that of the wild type (Fig. S5D–F), which provides indirect evidence for this assumption (Fig. 5D).

Since site Y was considered Br^−^ binding site rather than Cl^−^ binding site in the previous paper^33^, we conducted experiments where NaBr with or without L-Gln (200 µM) was applied to the cells maintained in 10 mM Cl^−^ buffer without changing the gluconate concentration before and after ligand application (see *Materials and methods* as well as Supplemental Table S3). Similar to the application of NaCl with or without L-Gln to mfT1R2a/mfT1R3 expressing cells (Fig. 3A), the responses of mfT1R2a/mfT1R3 to a fixed concentration of L-Gln were enhanced with an increase in the final Br^−^ concentration though NaBr alone did not elicit these responses (Fig. S6). This result further indicates that halogen acts as a PAM rather than an agonist in the concentration range used in this study, and also indicates the contribution of site Y, a previously considered Br^−^ binding site. As for the latter, however, it should be noted that the result may be affected by the replacement of Cl^−^ at site X by Br^−^ since the previous paper suggested that Br^−^ can also bind to site X^33^.

Contrary to our expectations, the critical residue (mfT1R2a K265) at site Y is not conserved in zfT1R2a, which showed susceptibility to Cl^−^ when coexpressed with zfT1R3 and applied with L-Pro (Figs. 1F and 3E). Furthermore, although mfT1R2b E260, which corresponds to mfT1R2a K265, was mutated to lysine or alanine to confer susceptibility to Cl^−^, the sensitivity of mfT1R2b E260K/mfT1R3 to L-Ala and L-Pro was hardly affected by [Cl^−^]_o_, similar to that of WT mfT1R2b/mfT1R3 and mfT1R2b E260A/mfT1R3 (Fig. S7). These results indicate that a single residue is not a determinative factor for Cl^−^ susceptibility of T1Rs.

## Discussion

Reportedly, many GPCRs are regulated by inorganic cations and anions^15,16^. In class C GPCRs such as CaSR and mGluRs, functional regulation by Cl^−^ has also been confirmed^20–22^. Regarding taste receptors, little is known about the issue, although the intraoral inorganic composition fluctuates in response to food and changes in the living environment. In this study, we investigated the role of extracellular Cl^−^ in the function of several T1Rs. Functional assays revealed that the sensitivity of some fish T1Rs was strikingly affected by [Cl^−^]_o_, indicating the generality of the functional modulation of class C GPCRs by Cl^−^.

In particular, the mechanism of the functional modulation of mGluRs by Cl^−^ has been characterized in detail^20,21^. In mGluRs, two Cl^−^ binding sites have been suggested: site 1, which is in the upper lobe of the VFTD, and site 2, which exists between the upper and lower lobes, except for mGluR2. Site 1 is widely conserved in class C GPCRs and corresponds to site X designated in this study within T1R3^8,22,33^. By comparing the results of site-directed mutagenesis of mGluRs, both sites 1 and 2 were confirmed to play a role in regulating the sensitivity of mGluRs by Cl^−20^. In our study, however, the sensitivity of hT1R2/hT1R3 to sucralose, D-tryptophan, and cyclamate and that of zfT1R2a/zfT1R3 to L-Ala was scarcely affected by [Cl^−^]_o_ in the range from 10 to 141.4 mM, although they also possess site X within the T1R3 subunit corresponding to site 1 (Figs. 1A,E and S1A,B). Notably, mfT1R2a/mfT1R3, mfT1R2b/mfT1R3, and mfT1R2c/mfT1R3 exhibited distinct susceptibility to [Cl^−^]_o_, although the mfT1R3 subunit was shared (Figs. 1, S1). Furthermore, prevention of Cl^−^ binding to site X did not notably alter the Cl^−^ susceptibility of T1Rs (Fig. 4). These results strongly indicated the negligible contribution of site X to the modulatory effect of extracellular Cl^−^, especially on T1Rs. This difference may be explained by the dimer composition, since mGluRs primarily form homodimers^35,36^, resulting in twice the number of site 1 per dimer. In contrast, T1Rs are predominantly heterodimeric receptors^2,8^, and site X is lacking in T1R1 and T1R2^33^, likely resulting in the weakened contribution of this site to susceptibility to Cl^−^. Notably, the lowest [Cl^−^]_o_ used in previous functional assays was 2 mM^20,21^, which is lower than that used in this study, and this may cause crucial differences. mGluR2 which lacks site 2 and only possesses site 1 (site X), however, exhibited lowered sensitivity even at 25 mM [Cl^−^]_o_^20^, indicating the difference in functional contribution of site X between mGluRs and T1Rs.

According to a recent report, on the other hand, Cl^−^ functions as an agonist of T1R2/T1R3 in various species by binding to site X within T1R3 rather than as an allosteric modulator^33^. In our study, while Cl^−^ enhanced the responses of mfT1R2a/mfT1R3 and zfT1R2a/zfT1R3 when coapplied with agonists, Cl^−^ alone did not elicit responses (Fig. 3A,B), indicating that Cl^−^ functions as a PAM. This discrepancy can be explained by different experimental conditions. First, owing to the instability of the cellular responses, we could not reduce the [Cl^−^]_o_ in the buffer to less than 10 mM. In the paper^33^, on the other hand, Cl^−^ was totally replaced with gluconate in biophysical experiments, and the tongues of mice were rinsed with distilled water before ligand applications in electrophysiological experiments. As binding of Cl^−^ to site X and its agonist-like action were observed at sub-mM to low-mM concentrationd^33^, most Cl^−^ was unlikely to be removed from site X even when cells were maintained in 10 mM Cl^−^ buffer in our experiments, explaining the lack of agonist-like function of Cl^−^ in this study. The role of Cl^−^ in T1Rs might differ depending on its concentration. Second, while they focused on the alterations in the thermostability and conformation of mfT1R2a/mfT1R3, we monitored T1R function through [Ca^2+^]_i_ fluctuations. Further investigation into the relationships between biophysical parameters and receptor function is needed.

In our study, the modulatory function of Cl^−^ on mfT1R2a/mfT1R3 was attributed to site Y, especially mfT1R2a K265, rather than to site X (Fig. 5A,B). However, only the binding of Br^−^, not Cl^−^, was confirmed at site Y in a previous crystallographic study^33^. This inconsistency may result from the weak interaction of Cl^−^ with site Y, as saturation of the sensitivity of mfT1R2a/mfT1R3 required 40–80 mM [Cl^−^]_o_ in our study (Fig. 2A,D). Interestingly, Br^−^ also acted as a PAM of mfT1R2a/mfT1R3 rather than an agonist (Fig. S6). Although 30 mM additional Cl^−^ was required to significantly increase the response intensity to L-Gln (Fig. 3A), 5 mM additional Br^−^ was sufficient to do so (Sup. Figure S6A). These results suggest that Br^−^ actually acts on mfT1R2a/mfT1R3 with a higher affinity. A recent study reporting that Br^−^ acted as a PAM of mGluR4 in a similar but more potent manner than Cl^−^ is also consistent with the results^37^.

Then, what is the molecular mechanism of action of Cl^−^ on mfT1R2a/mfT1R3 as a PAM? Since a biophysical study suggested that the conformational changes of mfT1R2a/mfT1R3 upon activation resemble those of mGluRs^38^, it is reasonable to refer to the action of Cl^−^ on mGluRs. The authors of the work focusing on rat mGluR3, which possesses site 2 at the interface of the VFTD lobes, proposed that Cl^−^ within the site may replace a neutral water molecule and create a stronger interaction with the residues in the upper and lower lobes of mGluR3 VFTD to stabilize the agonist-induced closed conformation^21^. Given that site Y, identified as critical for the modulation of the sensitivity of mfT1R2a/mfT1R3 by Cl^−^ in this study, is also located at the interface of the VFTD, a similar mechanism can be expected. Namely, only after agonists such as L-Gln and L-Ala occupy the cleft region within the VFTD of mfT1R2a^8^ to elicit the conformational shift of the VFTD into the closed active state, Cl^−^ may bind to site Y to bridge the K265 residue as well as the main-chain amide of K295 within the lower lobe and the main-chain amide of D362 within the upper lobe to stabilize the closed state (Fig. 5D). While Cl^−^ could solely interact with site Y, the binding of Cl^−^ alone would not cause the conformational shift of the receptor to activate the downstream signaling as evidenced in Fig. 3A even in such a case, which is contrary to the agonist-like action of the ion on site X at sub-mM to low-mM range^33^.

The sensitivity of zfT1R2a/zfT1R3 to L-Pro was also susceptible to Cl^−^ (Fig. 1F). As zfT1R2a/zfT1R3 and zfT1R2b/zfT1R3 showed susceptibility to extracellular anionic composition in different manners (Figs. 1 and 2), this susceptibility was likely attributable to the zfT1R2 subunit rather than the zfT1R3 subunit, which is shared by both heterodimers. Nevertheless, the residue corresponding to mfT1R2a K265 in zfT1R2a is proline, which is hydrophobic and unlikely to bind to the anion (Fig. 5E). The results indicate variable mechanisms that determine the PAM action of Cl^−^. Indeed, site 2 of the mGluRs is spatially distant from site Y^20^, which is important for the regulation of mfT1R2a/mfT1R3, as mentioned above (Fig. 5). Additionally, there is another Cl^−^ binding site located between the upper and lower lobes of the human CaSR VFTD and adjacent to site Y, which was called “site c” in a previous paper^22^. In summary, it is likely difficult to predict the susceptibility of class C GPCRs to Cl^−^ based solely on the primary sequence for each receptor. In fact, the introduction of a lysine residue into mfT1R2b E260, corresponding to mfT1R2a K265, did not confer susceptibility to Cl^−^ (Fig. S7).

As for the probe dependence of the susceptibility of zfT1R2a/zfT1R3, the lack of its structural information and the difficulty in predicting the key sites for Cl^−^ susceptibility based solely on the primary sequence makes it difficult to suggest reliable mechanisms on this issue. Given that Cl^−^ is considered to act as PAM of mfT1R2a/mfT1R3 and mGluRs except for mGluR2^20^ in a similar manner, i.e., binding to the interface of the upper and lower lobes of the VFTD to stabilize the closed conformation through the interaction with receptor-specific residues, it is plausible to assume that Cl^−^ functions in the same way when L-Pro is applied to zfT1R2a/zfT1R. If this is the case, the probe dependence of zfT1R2a/zfT1R3 can be explained by different conformations in its active closed forms depending on the agonist species, e.g. the proper distance between the upper and lower lobes to allow the Cl^−^ entry into the VFTD and stable interaction with those lobes may be maintained only under its L-Pro binding state. The chemical structure of L-Pro is unique compared to that of the other 19 natural α amino acids, including L-Ala, in that it is a cyclic amino acid. Such chemical structural differences may lead to distinct binding modes of zfT1R2a/zfT1R3 depending on the agonist applied, resulting in differences in closed conformation and Cl^−^ susceptibility. This hypothesis is an intriguing topic for future study.

Contrary to mfT1R2a/mfT1R3 and zfT1R2a/zfT1R3, zfT1R2b/zfT1R3 showed reduced sensitivity at lower [Cl^−^]_o_ conditions (Figs. 1D and 2C,F). However, no clear conclusion has been reached on whether Cl^−^ actually affects the function of zfT1R2b/zfT1R3 due to disturbance by osmotic pressure (Fig. 1F). Even if we assume that Cl^−^ actually inhibits the receptor function, predicting how Cl^−^ affects the function of zfT1R2b/zfT1R3 is currently difficult. One of the reasons for this is the lack of experimentally determined structure with information on ionic-binding sites, as in the case of zfT1R2a/zfT1R3, which makes it difficult to estimate the mechanism based on the molecular interaction between the ion and the specific sites within the receptor. Further structural studies are required to elucidate this point.

As for the physiological importance of the functional modulation of fish T1Rs by Cl^−^, fish taste receptors including mfT1R2a/mfT1R3 and zfT1R2a/zfT1R3 are likely used for feed searching. The sensitivity increase of these receptors depending on the extracellular concentration of Cl^−^ may contribute to more selective feeding of electrolyte-rich feed. Electrolyte uptake from the external environment is essential for freshwater fish to maintain their plasma osmolality. Medaka and zebrafish are omnivorous freshwater fish, feeding primarily on zooplankton and small benthos^39,40^. Chloride ion concentration in the hemolymph of freshwater amphipods is 60–80 mM^41,42^, and therefore, the greatly increased sensitivity of these T1R2s between 10 and 80 mM is beneficial for their feeding strategies in freshwater (Fig. 2D,E). However, the functional contribution of Na^+^ to T1Rs must be considered when discussing the roles of T1Rs in actual environments with various salinities. Although we tried to evaluate the functional contribution of Na^+^ to T1Rs by a Ca^2+^ mobilization assay, no clear conclusion could be drawn due to low reproducibility, presumably attributable to nonspecifically disturbed cellular function in buffers with low [Na^+^]_o_. Additionally, the lowest [Cl^−^]_o_ in this study was set at 10 mM because of response instability. Considering the reported agonist-like action of Cl^−^ on T1R3 at sub-mM to low-mM range^33^, we cannot rule out the possibility that there is also a regulatory action of Cl^−^ observed only in the range lower than 10 mM. Methodological improvements in the evaluation of the function of full-length T1Rs are required in future studies. The construction of an assay based on the conformational changes of full-length T1Rs, namely, time-resolved Förster resonance energy transfer (TR-FRET) assay^20–22,43,44^, is a promising strategy. Since the TR-FRET assay does not depend on overall cellular functions contrary to second messenger assays including Ca^2+^ mobilization assay, more reliable evaluation of the receptors’ function even under harsh conditions for cells, such as Cl^−^ free buffer, is expected with this method. We are currently working on the establishment of this novel assay.

In our previous study, mfT1R2c/mfT1R3 and zfT1R2a/zfT1R3 were identified as highly sensitive receptors for L-Pro^34^. In this study, their EC_50_ values for L-Pro were lower in 10 mM Cl^−^ buffer than in high Cl^−^ buffer (Fig. 1C,F), although only a modest or no effect of Cl^−^ on the sensitivity of mfT1R2c/mfT1R3 was suggested (Fig. S2). Notably, the modulatory effect of Cl^−^ on zfT1R2a/zfT1R3 varied depending on the agonist (L-Ala or L-Pro) as already described above (Fig. 1E,F). The L-Pro-specific Cl^−^-dependent sensitivity increase of zfT1R2a/zfT1R3 might cause the feed preference changes of zebrafish in response to environmental fluctuation; however, it needs further investigation of the effects of Cl^−^ on the taste receptors in a wider range of fish species including euryhaline fish in the same cyprinid family as zebrafish^45^ to know the biological significance of the relationship between L-Pro and Cl^−^ in taste preference.

In conclusion, extracellular Cl^−^ was confirmed to allosterically modulate the function of mfT1R2a/mfT1R3 and zfT1R2a/zfT1R3. While mfT1R2a K265 was found to be a key residue in this modulation, this residue was not conserved in zfT1R2a. Additionally, the susceptibility of zfT1R2a/zfT1R3 to Cl^−^ was agonist dependent, indicating a complicated mechanism for the functional regulation of T1Rs by Cl^−^.

## Materials and methods

### Sample solutions

The assay buffer (referred to as high Cl^−^ buffer) was composed of 10 mM 4-(2-hydroxyethyl)-1-piperazineethanesulfonic acid (HEPES), 130 mM NaCl, 10 mM glucose, 5 mM KCl, 2 mM CaCl_2_, and 1.2 mM MgCl_2_. The buffers with lower Cl^−^ were prepared by progressively replacing NaCl, KCl, CaCl_2_ and MgCl_2_ in the buffer with gluconate equivalents^20^. The pH was adjusted to 7.4 with NaOH. Ligands were diluted with the assay buffers at the desired concentrations.

Amino acids were obtained from commercial sources as follows. L-proline was purchased from Nacalai Tesque (Kyoto, Japan); L-alanine and L-glutamine were purchased from Kanto Chemical (Tokyo, Japan). Sucralose and sodium N-cyclohexyl-sulfamate (cyclamate) were purchased from Sigma–Aldrich Japan (Tokyo, Japan). D-tryptophan was purchased from Yoneyama Yakuhin Kogyo (Osaka, Japan).

### Evaluation of dose‒response relationship by cell-based assay

Measurements of the responses of the cells by cell-based assay based on the intracellular Ca^2+^ mobilization were carried out as described previously with some modifications^20,34,46,47^. Cell lines stably expressing T1R2, T1R3 and chimeric G-protein α-subunit, G16gust44^48^, were constructed using the Flp-In pcDNA5/FRT complete system (Thermo Fisher Scientific, Waltham, MA, USA) described in our previous studies^34,46^. The cells were seeded in 96-well plates (clear-bottomed CellBIND surface plate, Corning, Glendale, AZ, USA) coated with ε-poly-L-lysine (Cosmo Bio, Tokyo, Japan) at approximately 70,000 cells per well. After 20 h, the medium was removed and replaced with high Cl^−^ buffer. After 3 h 30 min incubation at 27 °C, cells were loaded with 5 µM calcium-indicator dye Fluo-8 AM (AAT Bioquest, Sunnyvale, CA, USA) diluted in high Cl^−^ buffer. After incubation for 30 min at 27 °C, the cells were washed with buffers containing appropriate concentrations of Cl^−^. The cellular assay was initiated after 3–5 min of resting and was completed within 5 min. The measurements were made using FlexStation 3 (Molecular Devices, Sunnyvale, CA, USA) at 27 °C. Fluorescence changes (i.e., excitation at 485 nm and emission at 525 nm with a cutoff at 515 nm) were monitored at 2 s intervals. A 100-µL aliquot of assay buffer supplemented with 2 × ligands was added at 20 s, and scanning was continued for an additional 40 s. The response of each well was determined as ∆RFU (delta relative fluorescent units), which was calculated as (maximum fluorescent value)—(minimum fluorescent value). Except for the receptor with severely disturbed functions (Fig. S4), the response value, averaged from duplicate or triplicate at each concentration and then normalized by the mean response value against the highest concentration of ligands obtained in the high Cl^−^ buffer in each experiment, were treated as n = 1. Such normalized data from more than three independent experiments are reported as the mean ± standard error of the mean (SEM). Dose‒response fitting curves were calculated by Prism 9 (GraphPad Software, San Diego, CA) using nonlinear regression (log(agonist) vs. response—variable slope), based on the integrated data from the several independent experiments.

### Evaluation of the effect of anions, cations, and osmotic pressure on cellular responses

Cells were seeded in 96-well plates coated with ε-poly-L-lysine at approximately 70,000 cells per well. After 20 h, the medium was removed and replaced with high Cl^−^ buffer. After 2 h of incubation at 27 °C, cells were loaded with 5 µM calcium-indicator dye Fluo-8 AM diluted in high Cl^−^ buffer. After incubation for 2 h at 27 °C, the cells were washed with buffer containing 10 mM Cl^−^. Fluorescence changes were monitored at 2 s intervals, as described above. A 100-µL of the NaCl solution, Na-gluconate solution or NaBr solution to obtain the final ionic composition shown in Supplemental Tables S1, S2 and S3, with or without corresponding agonists (sucralose at a final concentration of 150 µM for hT1R2/hT1R3, 200 µM L-Gln for mfT1R2a/mfT1R3, 160 µM L-Pro for zfT1R2a/zfT1R3, or 2 mM L-Ala for zfT1R2b/zfT1R3), was added at 20 s, and scanning was continued for an additional 40 s. The response of each well was determined as ∆RFU, calculated as (maximum fluorescent value)—(minimum fluorescent value). Three independent experiments were conducted for each receptor, and representative results are shown as the mean ± SEM (n = 3 replicates). Statistical tests were conducted using KyPlot version 6.0 (KyensLab Inc., Tokyo, Japan). Statistical significance was set at *p* < 0.05.

### Supplementary Information

Supplementary Figures.

Supplementary Legends.

Supplementary Tables.

## Data Availability

The datasets used and/or analysed during the current study available from the corresponding author on reasonable request.

## Abbreviations

CaSR: Calcium-sensing receptor
GPCR: G protein-coupled receptor
mf: Medaka fish
mGluR: Metabotropic glutamate receptor
NAM: Negative allosteric modulator
PAM: Positive allosteric modulator
RFU: Relative fluorescent units
T1R: Taste receptor type 1
TR-FRET: Time-resolved Förster resonance energy transfer
VFTD: Venus flytrap domain
WT: Wild type
zf: Zebrafish
