# Supplementary figures and images for: Allosteric modulation of the fish taste receptor type 1 (T1R) family by the extracellular chloride ion
Source: Sci Rep. 2023 Sep 28;13:16348. doi: 10.1038/s41598-023-43700-y (PMC10539361; doi:10.1038/s41598-023-43700-y)

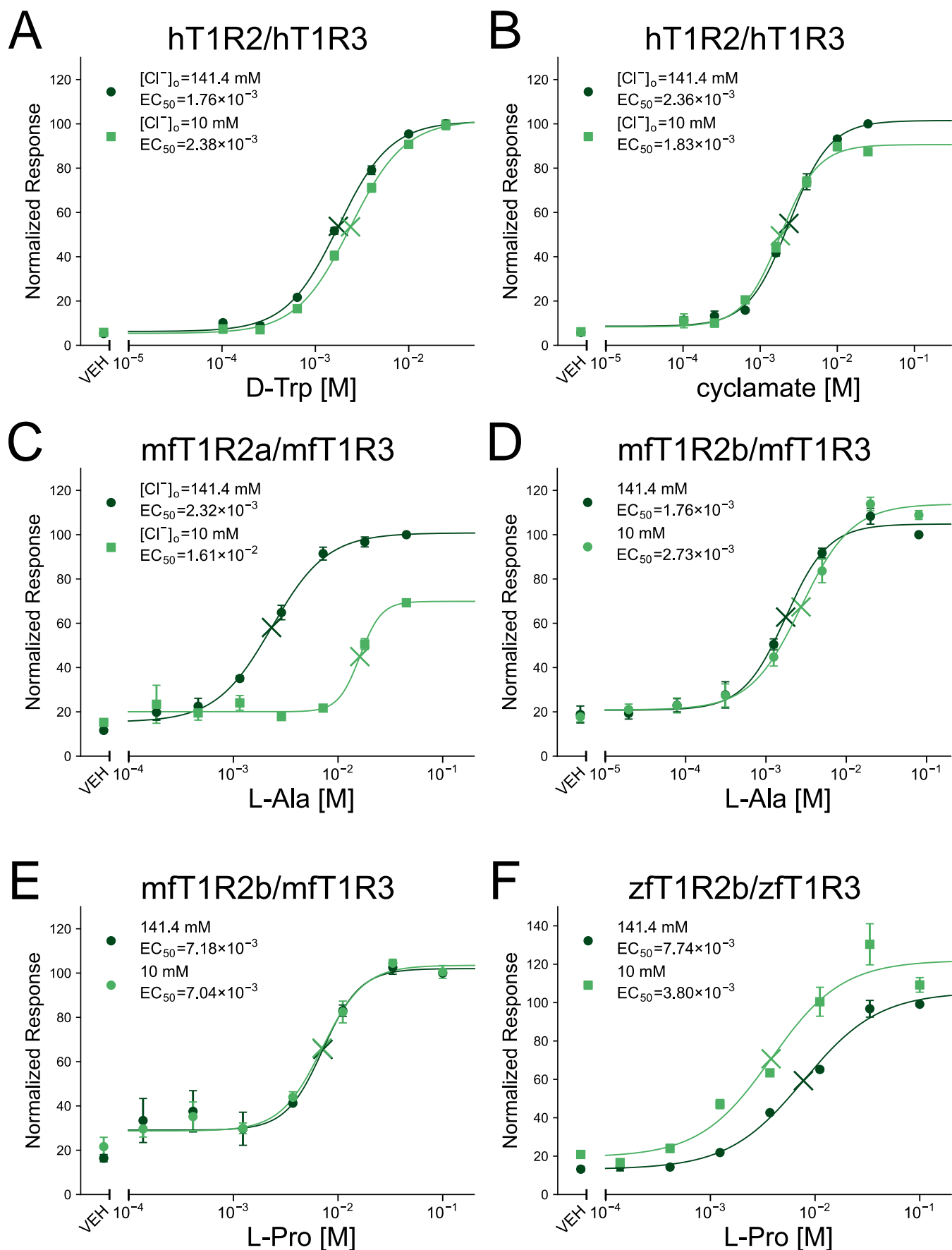

SupFig.1

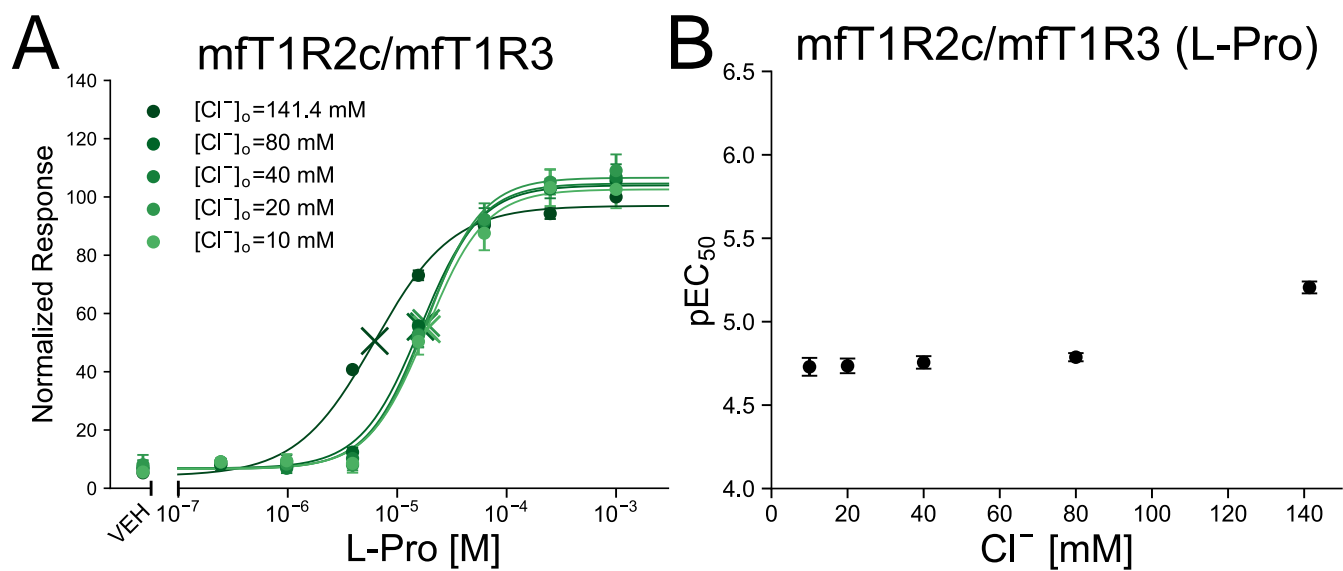

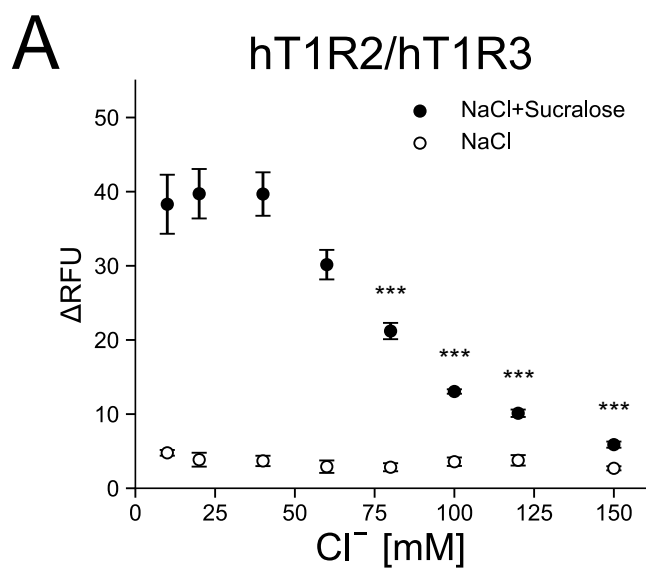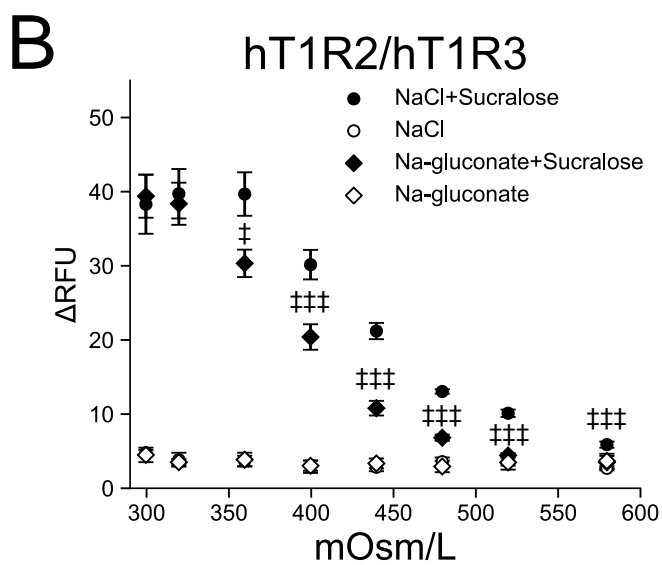

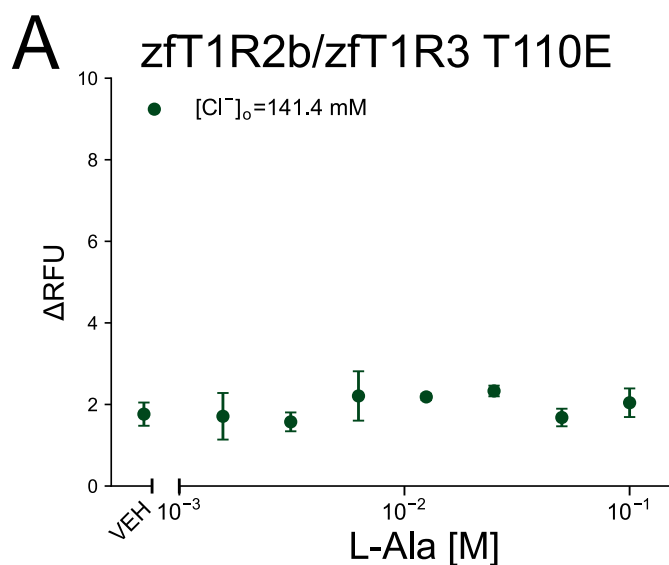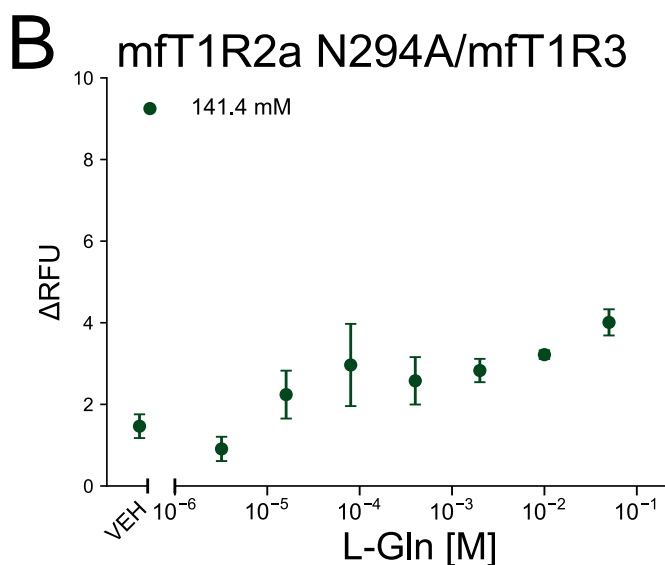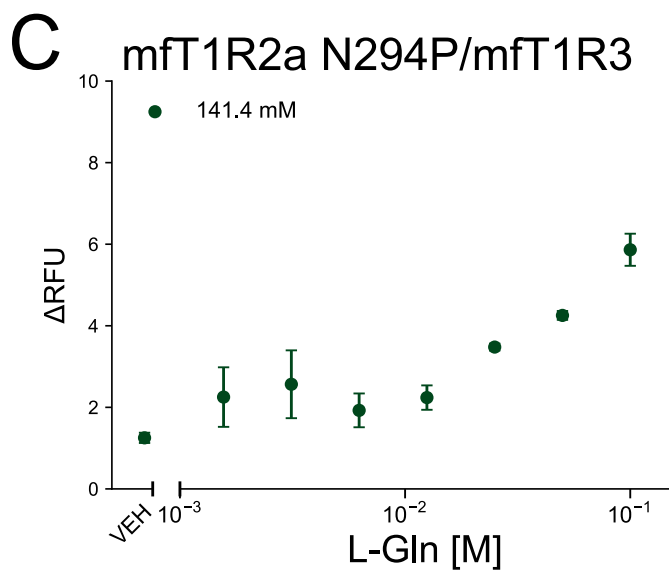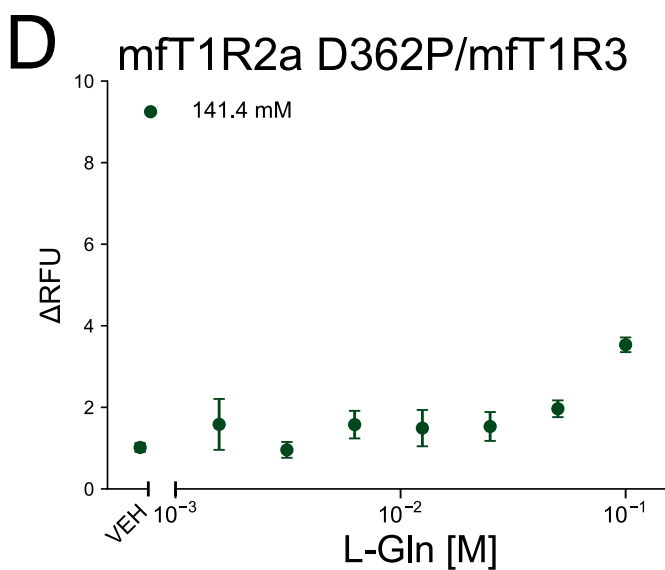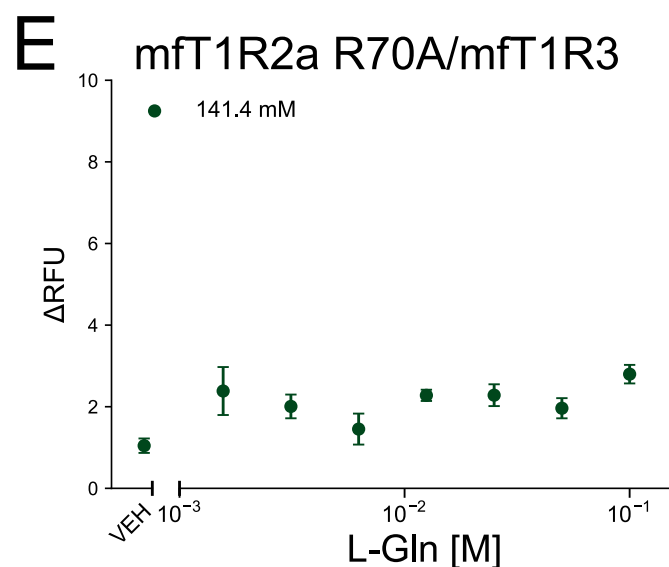

**A** mfT1R2a K295A/mfT1R3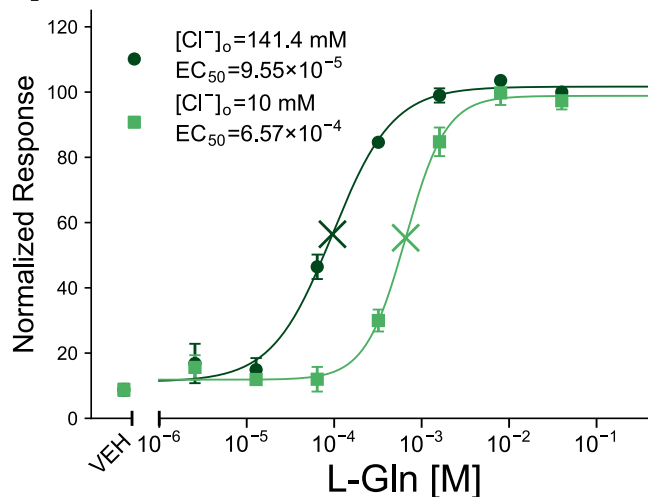**B** mfT1R2a K296P/mfT1R3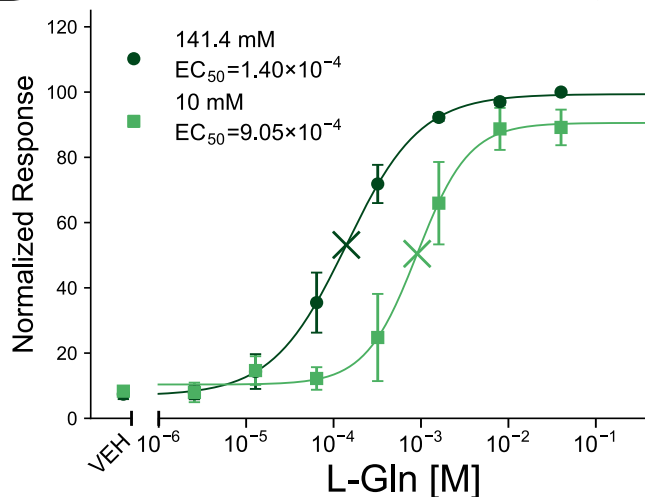**C** mfT1R2a K296A/mfT1R3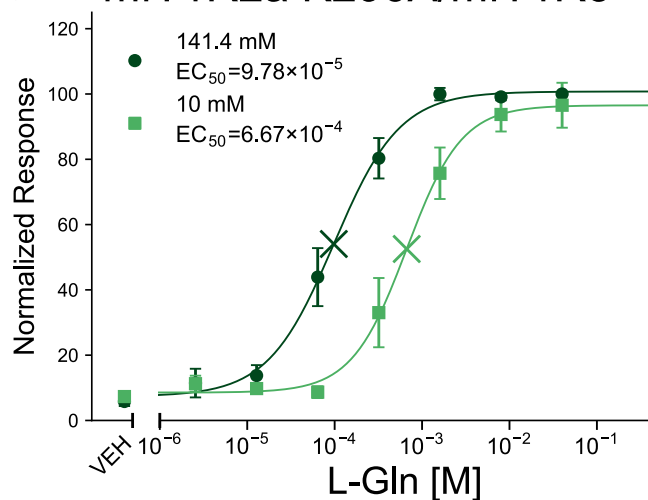**D** mfT1R2a L65P/mfT1R3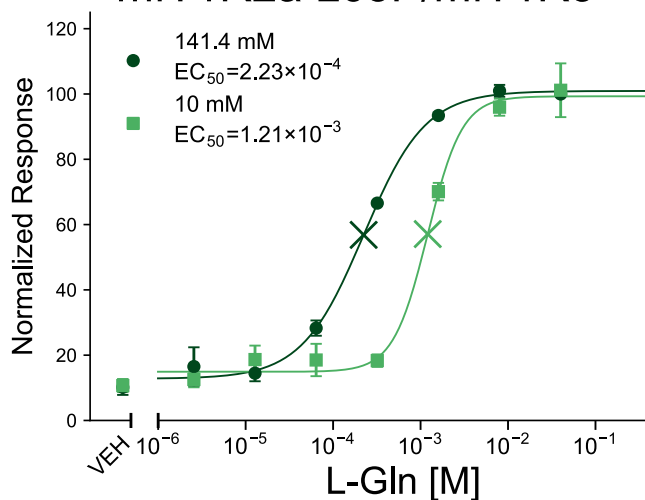**E** mfT1R2a N67A/mfT1R3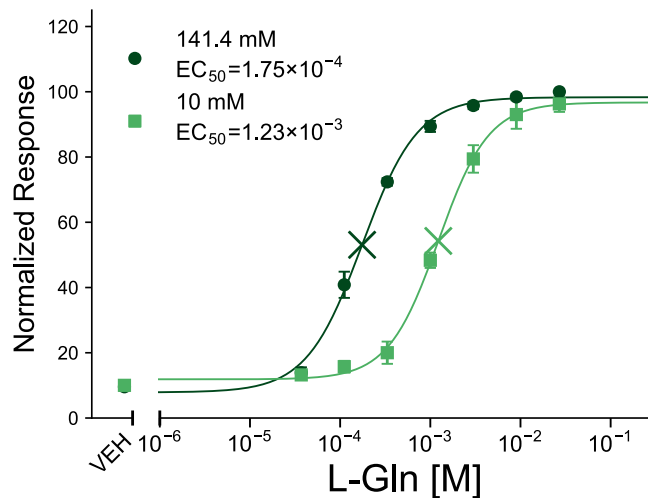**F** mfT1R2a D362A/mfT1R3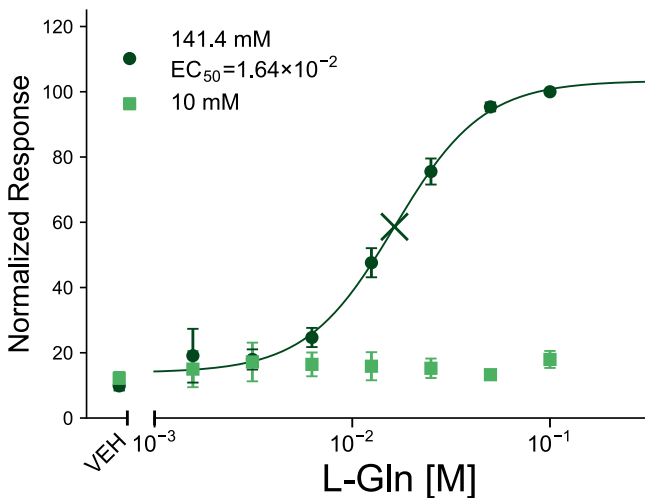

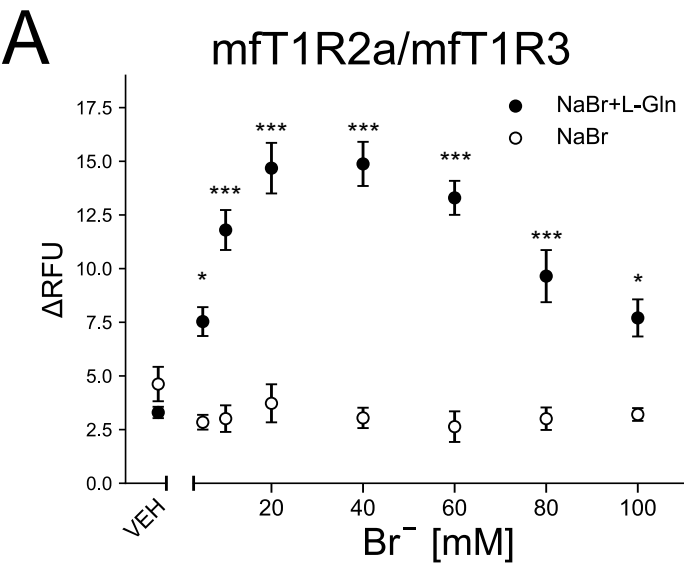

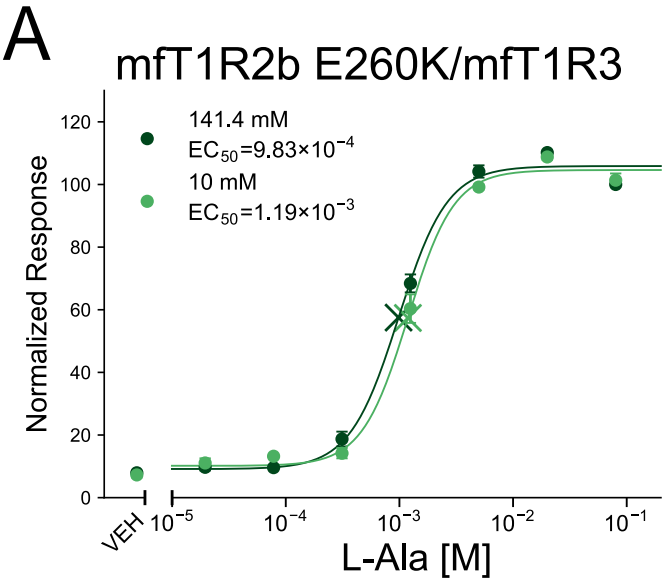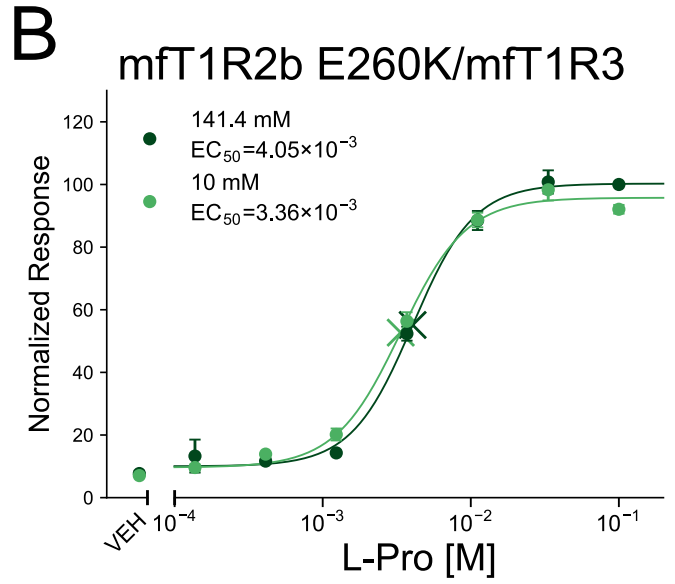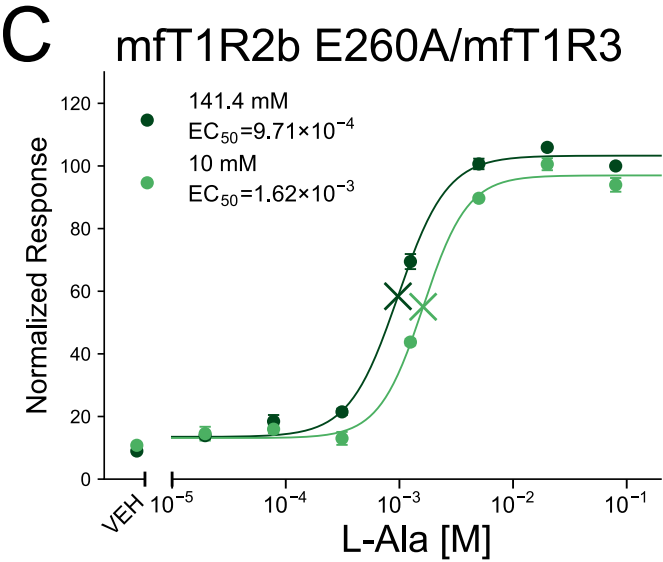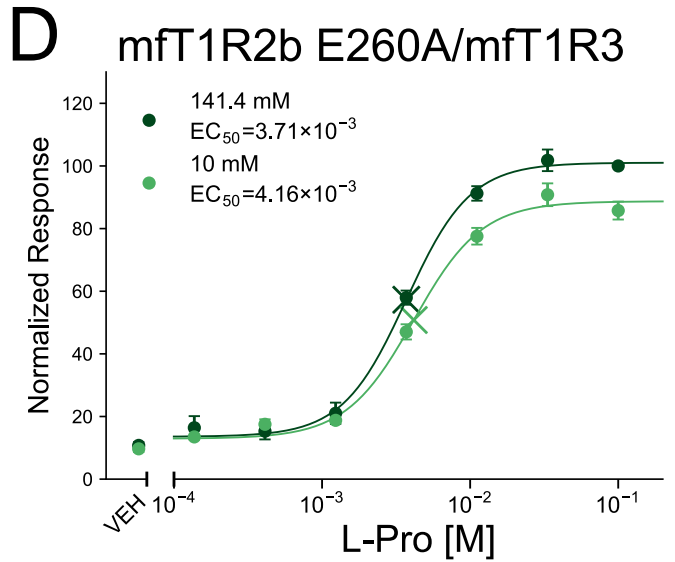

Supplement: Supplementary file 1 — Supplementary Figures. [file 41598_2023_43700_MOESM1_ESM.pdf]
