# Supplementary material for: Allosteric modulation of the fish taste receptor type 1 (T1R) family by the extracellular chloride ion
Source: Sci Rep. 2023 Sep 28;13:16348. doi: 10.1038/s41598-023-43700-y (PMC10539361; doi:10.1038/s41598-023-43700-y)
Supplement: Supplementary file 2 — Supplementary Legends. [file 41598_2023_43700_MOESM2_ESM.docx]

**Allosteric modulation of the fish taste receptor type 1 (T1R) family by the extracellular chloride ion**

Ryusei Goda^1^, Soichi Watanabe^2^, and Takumi Misaka^1,^ *

1. Department of Applied Biological Chemistry, Graduate School of Agricultural and Life Sciences, The University of Tokyo, Tokyo, Japan

2. Department of Aquatic Bioscience, Graduate School of Agricultural and Life Sciences, The University of Tokyo, Tokyo, Japan

^*^Correspondence to: Takumi Misaka

Graduate School of Agricultural and Life Sciences, The University of Tokyo, 1-1-1 Yayoi, Bunkyo-ku, Tokyo 113-8657, Japan.

Tel: +81-3-5841-8117; Fax: +81-3-5841-8100; E-mail: amisaka@mail.ecc.u-tokyo.ac.jp

**Fig. S1.** Different susceptibilities of T1Rs to extracellular anionic composition

Dose‒response curves of stable cell lines expressing (A) hT1R2/hT1R3 to D-Tryptophan, (B) hT1R2/hT1R3 to cyclamate, (C) mfT1R2a/mfT1R3 to L-Ala, (D) mfT1R2b/mfT1R3 to L-Ala, (E) mfT1R2b/mfT1R3 to L-Pro, or (F) zfT1R2b/zfT1R3 to L-Pro, with G16gust44. The raw data were normalized to the mean response against the highest concentration of ligands obtained in high Cl^-^ buffer in each experiment. Data are shown as the mean ± standard error of the mean (SEM, n=3 independent experiments performed in duplicate or triplicate). Cross marks in the graph represent EC_50_ values. VEH: responses against the buffer.

## **Fig. S2.** The relationships between extracellular Cl^-^ concentration and the sensitivity of mfT1R2c/mfT1R3

## (A) Dose‒response relationships of mfT1R2c/mfT1R3 to L-Pro obtained at 10 mM, 20 mM, 40 mM, 80 mM, and 141.4 mM [Cl^-^]_o_. Data are shown as the mean ± standard error of the mean (SEM, n=3 independent experiments performed in triplicate). (B) The negative logarithm of the EC_50_ values expressed in mol/L units (pEC_50_) calculated in (A) were plotted against [Cl^-^]_o_. Error bars indicate SEM. VEH: responses against the buffer.

## **Fig. S3.** The modulatory action of Cl^-^ applied to the cells maintained in 10 mM Cl^-^ buffer

(A) The responses of cells expressing hT1R2/hT1R3 with G16gust44. The cells were maintained in 10 mM Cl^-^ buffer and stimulated with NaCl solution to achieve the final ionic composition, as shown in Supplementary Table S1, with or without 150 μM sucralose. The responses were plotted against the final [Cl^-^]_o_ concentrations. (B) Na-gluconate solution was also applied to the cell to obtain the final ionic composition, as shown in Supplementary Table S2. The responses to Na-gluconate solution were plotted along with those to the NaCl solution, as shown in (A), against the final osmotic pressure. Three independent experiments were conducted, and representative results are shown as the mean ± SEM (n=3 replicates). Significant differences were analyzed using a one-way analysis of variance (ANOVA) followed by Dunnett’s test (****p* < 0.001 for vehicle vs NaCl + sucralose; ‡*p* < 0.05 and ‡‡‡*p* < 0.001 for vehicle vs Na-gluconate + sucralose). ∆RFU: Delta relative fluorescent units. VEH: responses against the 10 mM Cl^-^ buffer with or without sucralose.

## **Fig. S4.** T1R mutants with severely disturbed function

Dose‒response curves of stable cell lines expressing (A) zfT1R2b /zfT1R3 T110E to L-Ala, (B) mfT1R2a N294A/mfT1R3 to L-Gln, (C) mfT1R2a N294P/mfT1R3 to L-Gln, (D) mfT1R2a D362P/mfT1R3 to L-Gln, or (E) mfT1R2a R70A/mfT1R3 to L-Gln. Data are shown as the mean ± standard error of the mean (SEM, n=3 replicates). VEH: responses against the buffer.

## **Fig. S5.** The influence of mutation on residues around mfT1R2a K265 on the susceptibility of mfT1R2a/mfT1R3 to Cl^-^

Dose‒response curves of stable cell lines expressing (A) mfT1R2a K295A/mfT1R3, (B) mfT1R2a K296P/mfT1R3, (C) mfT1R2a K296A/mfT1R3, (D) mfT1R2a L65P/mfT1R3, (E) mfT1R2a N67A/mfT1R3, or (F) mfT1R2a D362A/mfT1R3 to L-Gln. Data are shown as the mean ± standard error of the mean (SEM, n=3-4 independent experiments performed in duplicate or triplicate). Cross marks in the graph represent EC_50_ values. VEH: responses against the buffer.

## **Fig. S6.** The modulatory action of Br^-^ applied to the cells maintained in 10 mM Cl^-^ buffer

(A)The responses of cells expressing mfT1R2a/mfT1R3 with G16gust44. The cells were maintained in 10 mM Cl^-^ buffer and stimulated with NaBr solution to achieve the final ionic composition, as shown in Supplementary Table S3, with or without 200 μM L-Gln. The responses were plotted against the final [Br^-^]_o_ concentrations. It should be noted that 10 mM of Cl- always existed in this experiment. Three independent experiments were conducted, and representative results are shown as the mean ± SEM (n=3 replicates). Significant differences were analyzed using a one-way analysis of variance (ANOVA) followed by Dunnett’s test (*p < 0.05 and ****p* < 0.001 for vehicle vs NaBr + L-Gln). ∆RFU: Delta relative fluorescent units. VEH: responses against the 10 mM Cl^-^ buffer with or without L-Gln.

## **Fig. S7.** Introduction of lysine residue into mfT1R2b E260 did not confer Cl^-^ susceptibility

Dose‒response curves of stable cell lines expressing (A) mfT1R2b E260K/mfT1R3 to L-Ala, (B) mfT1R2b E260K/mfT1R3 to L-Pro, (C) mfT1R2b E260A/mfT1R3 to L-Ala, or (D) mfT1R2b E260A/mfT1R3 to L-Pro. Data are shown as the mean ± standard error of the mean (SEM, n=3 independent experiments performed in duplicate). Cross marks in the graph represent EC_50_ values. VEH: responses against the buffer.
