# Supplementary material for: Allosteric modulation of the fish taste receptor type 1 (T1R) family by the extracellular chloride ion
Source: Sci Rep. 2023 Sep 28;13:16348. doi: 10.1038/s41598-023-43700-y (PMC10539361; doi:10.1038/s41598-023-43700-y)
Supplement: Supplementary file 3 — Supplementary Tables. [file 41598_2023_43700_MOESM3_ESM.pdf]

Supplemental Table S1. The final ionic composition after the application of NaCl solution

	Before	After (with or without agonist)							
Cl^- (mM)	10	10	20	40	60	80	100	120	150
gluconate (mM)	131.4	131.4	131.4	131.4	131.4	131.4	131.4	131.4	131.4
Na^+ (mM)	130	130	140	160	180	200	220	240	270
others (mM)	28.2	28.2	28.2	28.2	28.2	28.2	28.2	28.2	28.2
osmotic pressure (mOsm/L)	299.6	299.6	319.6	359.6	399.6	439.6	479.6	519.6	599.6

Supplemental Table S2. The final ionic composition after the application of Na-gluconate solution

	Before	After (with or without agonist)							
Cl^- (mM)	10	10	10	10	10	10	10	10	10
gluconate (mM)	131.4	131.4	141.4	161.4	181.4	201.4	221.4	241.4	271.4
Na^+ (mM)	130	130	140	160	180	200	220	240	270
others (mM)	28.2	28.2	28.2	28.2	28.2	28.2	28.2	28.2	28.2
osmotic pressure (mOsm/L)	299.6	299.6	319.6	359.6	399.6	439.6	479.6	519.6	599.6

Supplemental Table S3. The final ionic composition after the application of NaBr solution

	Before	After (with or without agonist)							
Cl^- (mM)	10	10	10	10	10	10	10	10	10
gluconate (mM)	131.4	131.4	131.4	131.4	131.4	131.4	131.4	131.4	131.4
Br^- (mM)	0	0	5	10	20	40	60	80	100
Na^+ (mM)	130	130	135	140	160	170	190	210	230
others (mM)	28.2	28.2	28.2	28.2	28.2	28.2	28.2	28.2	28.2
osmotic pressure (mOsm/L)	299.6	299.6	309.6	319.6	339.6	379.6	419.6	459.6	499.6
